# Mentalizing Self and Other and Affect Regulation Patterns in Anorexia and Depression

**DOI:** 10.3389/fpsyg.2019.02223

**Published:** 2019-10-15

**Authors:** Lily Rothschild-Yakar, Daniel Stein, Dor Goshen, Gal Shoval, Assaf Yacobi, Gilad Eger, Bar Kartin, Eitan Gur

**Affiliations:** ^1^School of Psychology, The Herta and Paul Amir Faculty of Social Sciences, University of Haifa, Haifa, Israel; ^2^Sackler Faculty of Medicine, Tel Aviv University, Tel Aviv, Israel; ^3^Sheba Medical Center, Ramat Gan, Israel; ^4^Geha Mental Health Center, Petah Tikva, Israel; ^5^Department of Psychiatry, Tel Aviv Sourasky Medical Center, Tel Aviv-Yafo, Israel

**Keywords:** mentalizing, alexithymia, theory of mind, affect regulation, eating disorders, depression

## Abstract

The study aimed to examine two constructs: general mentalizing processes and the specific component of affective mentalizing regarding self and others alongside the construct of affect regulation patterns in female adolescent and young adult inpatients with anorexia nervosa (AN; *n* = 41), depression (*n* = 20) and controls (*n* = 53). We further examined the predictive ability of affect regulation to eating-disorder (ED) symptoms beyond that of the mentalizing variables, and their potential role in mediating between mentalizing, depression and ED symptoms. We used tools assessing reflective functioning (RF), complex emotion recognition and theory of mind (ToM), alexithymia, affect regulation, depression, and ED symptoms. The AN and depression groups exhibited lower general mentalizing and higher alexithymia, emotional reactivity, and emotional cutoff patterns than controls, but showed no greater disturbance in ToM. The two clinical groups did not differ on any of these variables. Elevated mentalizing and adequate affect regulation patterns separately predicted lower severity of ED symptoms. Nonetheless, affect regulation did not add to the predictive value of mentalizing variables. Specifically, elevated alexithymia, and depressive symptomatology, but not RF, predicted greater ED symptomatology. Moreover, alexithymia directly accounted for elevated ED symptoms and also indirectly connected with ED symptoms via emotional hyperactivation and elevated depressive symptoms. These findings suggest that deficiencies in mentalization and affect regulation are not unique to AN, but may rather associated with psychopathology in general. Nonetheless, alexithymia and depression may increase ED-related symptomatology. Affect regulation deficiencies are mainly related with depressive symptoms and emotional hyperactivation is indirectly related with AN via the depressive symptoms.

## Introduction

Fonagy et al. (1998, 2002) suggested a model of mentalizing that provides a formulation of the normal development of modes of experience and of the development of psychopathology. Mentalizing refers to the capacity to reflect on and interpret one’s own behavior and that of others based on intentional internal mental states, such as beliefs, thoughts, and emotions (Fonagy et al., 1998, 2002). The ability to mentalize one’s own experiences and those of others allows for adequate coping with external and internal stressors, regulation of affects, and the formation of stable interpersonal relationships (Fonagy et al., 1998, 2002). Deficiencies in general mentalizing processes have been found in various psychopathologies, including depression, anxiety (Donges et al., 2005; Fischer-Kern et al., 2013), and EDs (e.g., Ward et al., 2001; Skårderud, 2007; Rothschild-Yakar et al., 2010).

The domain of mentalizing is multifaceted and includes four dimensions, each constructed of two poles (Fonagy and Luyten, 2009): (a) *explicit* vs. *automatic-implicit* mentalizing; (b) *cognitive* vs. *affective* mentalizing; (c) mentalizing based on *mental interiors* vs. *externally based* mentalizing; and (d) mentalizing *self-experience vs.* mentalizing the *experience of others.*

The first aim of this study was to examine the dynamics of anorexia nervosa (AN) in the framework of the mentalization-based model (Fonagy et al., 2002). Moreover, as there is a substantial comorbidity of depression in patients with AN (Allen et al., 2013), it is important to examine whether the deficiencies in mentalizing seen in AN are attributed to the ED *per se*, or whether they are related, at least in part, to comorbid depression. In this context, we specifically sought to examine whether the general ability of mentalizing and the specific component of affective mentalizing and the polarity of mentalizing affective self-experience vs. emotion recognition in others are related to ED- and depressive symptoms in patients with AN compared to patients with depression and healthy controls.

For this purpose, we examined the sub-construct of affective mentalizing based on related concepts of *Theory of Mind (ToM)* and *alexithymia*. Our approach is in line with that suggested by Luyten et al. (2012) regarding the assessment of the polarities of self-other mentalizing along the specific pole of affective mentalizing according to the related concepts of *ToM* and *alexithymia*.

*ToM* refers to the ability to form representations about intentional internal mental states such as thoughts, feelings, and beliefs (Heavey et al., 2000). It comprises both cognitive and affective aspects. The cognitive aspect includes the understanding the beliefs of others, whereas the affective aspect includes the recognition of the emotions of others and empathizing with them (Fonagy and Luyten, 2009). In our study, we examine emotion recognition. In contrast to *ToM*, which refers to understanding the other, *alexithymia* refers to the subjective experience of the self.

*Alexithymia*, as conceptualized and operationalized by Bagby et al. (1994, 2009), is a construct encompassing difficulties in identifying subjective feelings, describing feelings of the self to others, as well as a stimulus-bound, externally oriented cognitive style.

### Mentalizing, Affective ToM and Alexithymia in Anorexia Nervosa and Depression

According to the mentalizing model, EDs are conceptualized as related to primitive mentalizing modes of experience (Skårderud, 2007). Accordingly, people with EDs fail to develop the ability to differentiate between physical and emotional states and between one’s own experiences and those of others. Moreover, they are unable to ascribe causality to self and interpersonal experiences. These deficiencies may lead to the use of bodily and ED-related symptoms as a concrete means of representing and enacting feelings and thoughts and as a means of regulating drives and emotions (Bleiberg, 2001).

Several studies examining mentalyzing ability with the Reflective Function (RF) Scale (Fonagy et al., 1998) have supported these suggestions, showing that patients with AN exhibit a less developed ability to mentalize experiences compared to individuals without EDs (Ward et al., 2001; Rothschild-Yakar et al., 2010). A recent study examining mental state reasoning from movies further found that AN is specifically associated with poorer emotional mental state inference, whereas their non-emotional mental state inference is largely intact (Brockmeyer et al., 2016).

Models of self-psychology and attachment describe a strong tendency among people diagnosed with AN to devote extreme efforts to the identification and fulfillment of the needs of others, while rejecting their own needs (Wechselblatt et al., 2000; Bachar et al., 2010). Along these lines, patients with AN present with elevated levels of *alexithymia* compared to patients with bulimia nervosa (BN) and controls (Corcos et al., 2000; Speranza et al., 2005). The findings are less consistent when assessing emotion recognition of *others (ToM).* Thus, deficits in emotion recognition of others have been found in some studies of patients with AN (Harrison et al., 2009), but not in others when applying *ToM* measures (Bentz et al., 2017) or other paradigms (e.g., Wyssen et al., 2019).

The few studies examining RF in patients with depression yield conflicting results. Fischer-Kern et al. (2013) found low RF scores in female inpatients with major depressive disorder (MDD) compared to a control sample, whereas Taubner et al. (2011) did not find a difference between patients with chronic depression and healthy controls. Regarding the polarity of self-other mentalizing, theories explaining depressive disorders according to the mentalizing model emphasize an increased preoccupation with the self in depressed patients (Donges et al., 2005) and a lower ability to handle complex emotions of others (Bateman and Fonagy, 2015). Moreover, people with depression are preoccupied with self-experience, are less empathically attuned to others, and have greater deficiencies in their ability to conceptualize emotions of others but not their own (Donges et al., 2005). In addition, patients with depression exhibit more reflective statements regarding the self than control participants, whereas no between-group differences have been found in the rate of reflective sentences about others (Taubner et al., 2011).

A meta-analysis comparing patients with MDD to patients with anxiety disorders has found that although both groups show difficulty in recognizing the emotions of others, individuals with depression show greater deficiency in *ToM* than those with anxiety disorders (Demenescu et al., 2010).

Research has shown a consistent association between *alexithymia* and depression (Taylor and Bagby, 2004). Several longitudinal studies have shown that a change in *alexithymia* predicts a change in depressive symptoms over time (e.g., Honkalampi et al., 2001). Nonetheless, other studies point to a relative stability of *alexithymia* in patients with depression, even following a reduction in the severity of depressive symptoms (Luminet et al., 2001).

A developed ability to mentalize is considered a key factor in the development of adequate affect regulation abilities (Fonagy et al., 2002). Nonetheless, other scholars propose an opposite directionality, suggesting that cognitive deficit and neuropsychological proneness of emotion dysregulation may affect the development of mentalizing and reflective abilities (Bleiberg, 2001). This has led us to refer to affect regulation and mentalizing as distinct processes that are closely interrelated. Accordingly, we have sought to examine the predictive ability of each construct (mentalizing and affect regulation) on the severity of ED symptoms, and the potential role of affect regulation in mediating between mentalizing, depression and ED symptoms.

#### Affect Regulation in Eating Disorders and Depression

A developed ability to regulate affects involves the ability to cope with distress and attenuate negative emotions, alongside strengthening positive emotions (Schore, 2001). Faulty affect regulation is considered important in the predisposition to and maintenance of EDs (Gilboa-Schechtman et al., 2006) and participants with EDs have been found to present with deficient affect regulation strategies, specifically with elevated use of emotion suppression and lower cognitive reappraisal than controls (Rothschild-Yakar et al., 2018).

Depression may be also associated with a dysfunction of cognitive and emotion regulation strategies (e.g., Mennin et al., 2007). There is also a strong link between depression and irritability, and chronic severe emotional dysregulation in childhood and adolescence may predict later depression (Vidal-Ribas et al., 2016, 2018).

Adequate emotion regulation is based on the ability to distinguish thoughts from emotions and to be guided by one’s own intellect and emotions rather than by those of others (Bowen, 1978). Thus, poorly differentiated people tend to be more emotionally reactive and/or to cut themselves off emotionally when facing emotionality of others. Emotional reactivity (ER) is regarded as the degree to which an individual respond with emotional flooding, lability, or hypersensitivity to anxiety aroused by relational distress. Emotional cutoff (EC), on the other hand, is regarded as emotional distance, fear of intimacy and/or suppression of emotions (Kerr and Bowen, 1988). Studies have shown that both dysfunctional emotion regulation patterns, may be associated with a greater predisposition to both depression (Bowen, 1978; Choi and Murdock, 2017), and EDs (Tasca et al., 2009; Rothschild-Yakar et al., 2016).

#### Study Aims and Hypothesis

The first aim of the study was to compare general mentalizing ability, specific affective mentalizing along the self-other axis, and affect regulation patterns in patients with AN, depression and healthy controls. In light of the findings for mentalizing, alexithymia and affect regulation, we have specifically decided to include a comparison group with depression, to find out whether the differences in mentalizing abilities and affect regulation are specific to AN, or will be rather associated with the depressive component.

Our second aim was to examine the role of mentalizing, affect regulation patterns and depression in predicting the severity of ED symptoms in AN. We were specifically interested to examine whether affect regulation patterns may predict the severity of ED symptoms in AN above and beyond the influence of the mentalizing variables. Our third aim was to explore whether mentalizing variables are directly related to the ED symptoms or mediated by affect regulation strategies and depressive symptoms.

We hypothesized that: (1) the two clinical groups would show lower RF, lower ability to identify the emotions of others (*ToM*), greater *alexithymia* and deficient patterns of affect regulation than the control group. We further anticipated that patients with AN would show greater deficiency in mentalizing the affective experience of the self (*alexithymia*) than patients with depression, whereas participants with depression would exhibit a lower capacity for mentalizing of the other (*ToM*) than patients with AN; (2) lower levels of RF and *ToM* and higher levels of *alexithymia* would predict more severe ED-related symptoms in patients with AN. Moreover, *alexithymia* would be the strongest predictor of ED severity. (3) Deficient affect regulation patterns would predict more severe ED-related symptoms. (4) Affect regulation patterns and depression would predict the severity of ED symptomatology above and beyond the influence of mentalization. (5) Affect regulation patterns and depressive symptoms will serve as mediating factors between mentalization and ED symptoms.

## Materials and Methods

### Participants

The AN sample comprised of 41 female inpatients between the ages of 14–22 years (*M* = 17.58 ± 2.57) hospitalized in the adolescent or adult ED inpatient departments at the Sheba Medical Center, Tel Hashomer, Israel. All patients met the DSM-5 (American Psychiatric Association (APA), 2013) criteria for diagnosis of AN on admission and had no lifetime or current diagnosis of bipolar disorder, schizophrenia spectrum disorder, substance use disorder, mental retardation, organic brain syndrome, and any physical disorder except for inter-current medical problems. The patients were diagnosed with either AN-restricting type (AN-R; *n* = 29) or AN binge/purge type (AN-B/P; *n* = 12). Eighteen patients (44%) were diagnosed with comorbid depressive disorders, three (7%) with comorbid anxiety disorders, and 6 (14%) with obsessive compulsive disorder (OCD).

The second group included 20 adolescent female patients between the ages 14–20 years (*M* = 15.85 ± 1.85) diagnosed with affective disorders according to the DSM-5 (American Psychiatric Association (APA), 2013), including major depressive disorder and dysthymic disorder (*n* = 19) and bipolar disorder in a depressive episode (*n* = 1). These patients were hospitalized either in the adolescent day center or the adolescent inpatient department of the Geha Mental Health Center, Rabin Campus, Petah Tikva, Israel. Comorbid diagnoses in these patients were anxiety disorders (*n* = 5, 25%), oppositional defiant disorder (*n* = 1, 5%) and OCD (1, 5%). Exclusion criteria were a history of lifetime or current schizophrenia spectrum disorder, substance use disorder, mental retardation, organic brain syndrome, EDs, body mass index (BMI) ≤ 17 kg/m^2^, and any lifetime or current physical disorder except for inter-current medical problems.

The control sample comprised of 53 female high school and undergraduate students between the ages 14–22 years (*M* = 17.63 ± 2.39) volunteering to participate in the study, and other undergraduate students participating as part of the requirements for completing their degree. Controls were required to have no lifetime or current history of any psychiatric illness, no physical illness except for inter-current medical problems, no chronic use of medications (defined as not using medications for at least four consecutive weeks), and no stigmata indicative of an ED [i.e., BMI between 18.5–25 kg/m^2^ (Bray, 1992), menstruation was regular from menarche unless using birth control pills, and there was no evidence of pathological eating-related behaviors]. Four control participants were excluded from the study because of failing to fulfill the above-mentioned criteria.

To control for intellectual ability, we administered two subtests from the Hebrew edition of the Wechsler Adult Intelligence Scale (WAIS-III-Heb; Wechsler, 1997; WISC-IV Heb; Wechsler, 2004) as an estimate of Intelligence Quotient (IQ): block design and similarities. Estimated IQ was the average score on these two subtests.

### Instruments

#### Psychiatric Diagnosis

##### AN group

Diagnosis of AN and of comorbid psychiatric diagnoses, and the lack of the excluded psychiatric diagnoses in the AN group were established using the Structured Clinical Interview for DSM-IV Axis I Disorders-Patient Edition (SCID-I/P Version 2.0) (First et al., 1995). The diagnoses achieved with the SCID-I/P Version 2.0 were adapted for the DSM-5 diagnosis of AN.

##### Depression group

Diagnosis of DSM-5 (American Psychiatric Association (APA), 2013) affective disorders and other comorbid psychiatric disorders and the lack of the excluded psychiatric disorders in the depression group was achieved using structured clinical interviews based on the criteria of the Schedule for Affective Disorders and Schizophrenia for school-age children-present and lifetime version (K-SADS-PL) (Kaufman et al., 1997).

The participants with depressive disorders were further screened for ED-related symptoms using the SCOFF (Perry et al., 2002). Answering positively on two of the five items of the SCOFF was found to have excellent validity in differentiating people with problematic eating-related behaviors from non-ED individuals (Perry et al., 2002). In the present design, we excluded control participants answering positively on any one item of the SCOFF.

Three participants in the depression group received a SCOFF score higher than 2. Nonetheless, as they were not diagnosed with an ED using the structured clinical interviews, we decided to include them in the study [note that there may be an incidence of 12.5% false positive diagnoses of possible eating-related disturbances using the SCOFF (Morgan et al., 1999).

Both clinical groups were treated with anti-depressive medications when assessed.

##### Control group

Control participants were interviewed using the ten general screening criteria of the SCID-I/P Version 2.0, as well as the specific screening items for affective disorders and schizophrenic spectrum disorders. Each screening item of the SCID-I/P Version 2.0 is rated as either present (positive), questionable, or not present (negative). Only those answering negatively on all SCID- I/P Version 2.0 screening items were included as controls in the study. For a similar approach, see Stein et al. (2002) and Cardi et al. (2013).

Control participants were further screened for ED-related symptoms using the SCOFF interview (Perry et al., 2002), similar to its use in the depression group.

#### Mentalizing

##### The Reflective Function (RF) Scale (Fonagy et al., 1998)

The RF scale integrates the assessment of multiple facets into a global rating of the quality of mentalizing in the specific context of childhood attachment relationships via narratives derived from the Adult Attachment Interview (AAI; George et al., 1996). Interviews are scored on an 11-point rating scale, ranging from −1 (negative RF, in which interviews are overly concrete or grossly distort others’ mental states); through ordinary RF that is common in non-clinical populations, to +9 (exceptional RF, in which interviews show unusually complex, elaborate, or original reasoning about mental states of self and others).

Three coders trained by the first author, who had been trained by the developers of the coding manual, scored the RF protocols. One coder scored all the protocols. Two coders who were blind to the participants’ group allocations coded a subset of the transcripts (*n* = 33 in ED patients and controls, and *n* = 10 in patients with depression), yielding excellent inter-rater reliability, intraclass correlation coefficient (ICC) for RF = 0.94.

##### Complex Emotion Recognition and Theory of Mind – Reading the Mind in the Eyes Task (RME) (Baron-Cohen et al., 2001)

The task was designed to assess the ability to recognize basic and complex emotions in others. Participants were shown 36 photographs of eyes expressing emotions and were asked to make a forced decision between four words naming the emotion (one correct and three distractors). There was no time limit for this task, but participants were asked to complete it as quickly as they could.

##### TAS-20 – The Toronto Alexithymia Scale (Bagby et al., 1994)

The TAS represents the subjective assessment of difficulties in recognizing internal emotions of the self. The 20 items of the TAS-20 are divided into three subscales: difficulty in identifying feelings (DIF, seven items), difficulty in describing feelings (DDF, five items) and externally oriented thinking (EOT, eight items). Participants rate items on a 6-point scale ranging from strongly disagree (1) to agree (6). Cronbach’s alpha in this study for the full scale = 0.88; for DIF = 0.88, for DDF = 0.87, and for EOT = 0.61.

#### Affect Regulation

##### DSI- Self-reported differentiation of self

The Differentiation of Self Inventory (DSI; Skowron and Friedlander, 1998; Skowron and Schmitt, 2003) is a self-report instrument for adults (age ≥ 25 years), that was modification and validated for adolescents and young adults (Rothschild-Yakar et al., 2016). We have used in this study two subscales of affect regulation from Skowron and Schmitt’s (2003) revised version: (1) The 11-item ER subscale reflects the degree to which a person responds to environmental stimuli with emotional flooding, lability or hypersensitivity. (2) The 12-item Emotional Cutoff subscale (EC) reflects feeling threatened by intimacy and suppression of emotions. Higher scores in both scales represent greater affect regulation ability. Participants rate items on a 6-point Likert-type response scale ranging from *not at all* (1) to *very true* (6). Internal consistencies in the current study: ER = 0.83 and EC = 0.84.

#### Depression

##### Beck Depression Inventory (BDI)

Beck Depression Inventory (BDI) (Beck, 1961) is a 21-item self-report assessing the severity of depressive symptoms at the time of the evaluation. Participants rate items on a 4-point scale ranging from rarely (1) to often (4). Cronbach’s alpha for the BDI in this study = 0.95.

#### ED Symptoms

##### The Eating Attitudes Test (EAT- 26)

The Eating Attitudes Test (EAT- 26) (Garner et al., 1982) is a self-report scale assessing concerns and behaviors related to eating. Participants rate items on a 6-point scale ranging from never (1) to always (6). Cronbach’s alpha of the EAT-26 in this study = 0.96.

##### The SCOFF- questionnaire for the assessment of eating disorders

The SCOFF- questionnaire for the assessment of eating disorders (Morgan et al., 1999) includes five yes/no questions developed to screen for eating disorders in the general population. One point is given for every positive answer. A total score of two and above has been found to be 100% sensitive and 87.5% specific for the presence of pathological eating and weight-related behaviors (Morgan et al., 1999).

Finally, demographic and clinical variables, including age, education level, and losses in the family, were recorded using a demographic questionnaire and from the patients’ medical records.

### Procedure

All participants, and their parents or other legal guardians in the case of minors under the age of 18, gave their written informed consent to participate in the study after receiving an explanation of the study’s goals and methodology. The study was approved by the Internal Review Boards (Helsinki Boards) of the Sheba Medical Center, Tel Hashomer, and the Geha Mental Health Center, Petah Tikva, Israel.

Both patients with AN and with depressive disorders were interviewed on admission by highly experienced certified psychiatrists and child and adolescent psychiatrists. Diagnoses were confirmed in clinical team meetings of the respective departments. Controls were interviewed with the screening criteria of the SCID-I/P Version 2.0 and the SCOFF by researchers trained with the use of these tools by a senior psychiatrist (DS).

The study measures were administered individually by four trained master’s level clinical psychology students in the ED departments and one trained master’s level clinical psychology student in the general psychiatric inpatient department and day center. The evaluators assessing the RF and RME tasks were blind to the self-report results. Patients in the two ED inpatient departments were assessed only after stabilization of their overall medical condition, as determined by physical examinations and relevant laboratory tests, to reduce the influence of their physical condition on the study findings. Standing height in both inpatient groups was measured to the nearest 0.1 cm, using a wall mounted stadiometer. All measurements were taken during the morning hours using standardized procedures (Tanner, 1994). Body weight was obtained to the nearest 0.1 kg, with the patient wearing a hospital gown and without any footwear. Control patients reported of their weight and height last, after filling-out all other questionnaires, to minimize the effect of weight on their responses.

### Data Analyses

Descriptive statistics of the study variables were carried out through means and standard deviation (SDs) for the continuous variables and percentages for the categorical variable. To examine whether the two AN subtypes (AN-R and AN-B/P) differed in sociodemographic characteristics ED and depressive symptoms and the study variables we conducted multivariate analyses of variance (MANOVAs) models and chi-square tests. Then, a multivariate analyses of variance (MANOVA) model and chi-square tests were applied to examine the differences between the study groups (AN, depression and controls) in sociodemographic characteristics. Subsequently, age and parents’ education served as covariates in all the remaining analyses. We further applied a *t*-test to examine the difference between the clinical groups in the duration of illness before hospitalization.

Between group differences in mentalizing variables and affect-regulation patterns were assessed by a multivariate analysis of covariance (MANCOVA), using Wilk’s Lambda criterion. *Post hoc* comparisons were conducted using Tukey–Kramer adjustment.

The associations between the study measures were tested using partial Pearson Correlation coefficients. A series of three hierarchical linear regression analyses were performed in order to evaluate the unique and mutual contribution of mentalizing, affect regulation patterns, and depressive symptoms for the prediction of ED symptoms.

To examine the potential mediating role of affect-regulation patterns and depressive symptoms in the association between mentalizing variables and ED symptoms mediation analyses were applied. The analyses were conducted with Hayes and Preacher guidelines (Preacher and Hayes, 2008) using the PROCESS macro (v.3.3; Hayes, 2018), which enables testing of multiple paths while employing a bootstrapping procedure with 10,000 resamples. All variables and covariates in the mediation model were converted into *z*-scores (with mean of zero and standard deviation of one). In this analysis 114 subjects were used. According to bootstrap simulations (Fritz and Mackinnon, 2007) a simple mediation model with 115 subjects can detect a mediation path [(1−β) = 0.8] assuming that the path X→M_1_ ≥ 0.59 and M_1_→Y ≥ 0.26. According to Fritz et al. (2015) adding a second mediator to the model which is moderately correlated (0.39) with X would decrease the power of the model by −0.040 to 0.058, assuming that the path M_2_→Y = 0.14 and by −0.056 to 0.072 assuming that M_2_→Y = 0.39. Thus, we estimated that the power of the multipath model analysis is about 75% for one significant mediator and 73% for two significant mediators.

Statistical analyses were performed using SAS software for windows, version 9.4, Cary, NC, United States: SAS Institute; statistical significance was set at 0.05.

## Results

A MANOVA and a chi-square test comparing the two ED subtypes in sociodemographic variables revealed no significant differences for age, estimated IQ, parents’ education, BMI, ED and depressive symptoms and family lose [Wilk’s Lambda *F*_(__8__,__3__2__)_ = 0.35, n.s; χ^2(1)^ = 0.85, n.s; respectively]. No significant difference emerged for mentalizing abilities with the dependent variables: general RF, affective ToM (RME) and alexithymia (TAS) [Wilk’s Lambda *F*_(__3__,__3__7__)_ = 0.86, n.s]. No significant between-group difference emerged also for affect regulation patterns [Wilk’s Lambda *F*_(__2__,__38__)_ = 0.52, n.s.]. The lack of differences in the sociodemographic, symptomatic and study measures between the two ED subgroups allowed us to relate to all patients with AN as belonging to one group.

Table 1 compares the demographic characteristics of the three study groups using analyses of variance (ANOVAs). The data revealed significant differences for age, mother’s and father’s educational level, and BMI. Thus, we controlled for age and mother’s and father’s educational level in the statistical analyses using analyses of covariance (ANCOVAs). In addition, no differences were found in duration of illness until hospitalization between patients with AN (4.12 ± 2.8 months) and patients with depression (4.00 ± 1.5 months; *t* = 0.18; *p* = 0.86).

**TABLE 1:** Demographic and clinical variables in patients with EDs, affective disorders, and controls.

**Variables**	**Patients with Anorexia Nervosa (*n* = 41)**	**Patients with depression (*n* = 20)**	**Non-patient controls (*n* = 53)**	***F* (2,111)**	***P*-value**	**η^2^**
	***M* ± *SD***	***M* ± *SD***	***M* ± *SD***			
Age	17.58 ± 2.57	15.85 ± 1.85	17.63 ± 2.39	4.52	*p* = 0.03	0.075
Mother’s education	14.02 ± 2.43	15.25 ± 4.12	15.39 ± 2.19	3.15	*p* = 0.03	0.054
Father’s education	13.34 ± 2.32	14.35 ± 2.92	15.34 ± 2.14	8.34	*p* < 0.001	0.13
Losses in family	9.8%	5%	1.9%	1.44	*p* = 0.25	0.03
Body mass index	18.04 ± 1.72	21.90 ± 4.35	21.21 ± 2.46	21.14	*p* < 0.001	0.28
Estimated IQ	10.84 ± 1.99	10.40 ± 2.11	11.55 ± 1.96	2.70	*p* = 0.07	0.05

*EDs, eating disorders.*

Table 2 shows the between-group differences in ED symptoms and depression. Tukey–Kramer’s *post hoc* comparisons of the three groups showed that patients with AN reported significantly more ED symptoms (EAT-26) than patients with depression and controls. The two clinical groups reported significantly higher levels of depressive symptoms than the control group, but the two groups did not differ from each other.

**TABLE 2:** Two-way analyses of variance of the study variables for the three groups.

**Variable**	**A Patients with AN (*n* = 41)**		**B Patients with depression (*n* = 20)**		**C Non-patient controls (*n* = 53)**		***F* (2,108)**	***P*-value**	**η^2^**	**Tukey–Kramer *post hoc***
	***M***	***SD***	***M***	***SD***	***M***	***SD***				
EAT-26	49.16	14.73	16.03	15.33	9.91	8.81	115.14	*p* < 0.001	0.68	A > B^∗∗∗^,C^∗∗∗^
BDI	33.42	13.07	33.45	18.65	5.50	8.63	68.35	*p* < 0.001	0.56	C < A^∗∗∗^,B^∗∗∗^
General RF	3.53	1.53	3.71	1.56	4.69	1.30	7.49	*p* < 0.01	0.12	C > A^∗∗^,B^∗^
RME	24.57	3.95	24.56	5.33	24.98	3.13	0.14^a^	*p* = 0.74	0.00	
TAS-20	64.10	12.28	65.10	12.27	44.15	9.67	40.38^b^	*p* < 0.001	0.43	C < A^∗∗∗^,B^∗∗∗^
TAS dif	25.23	5.86	25.92	5.84	16.31	5.21	32.7	*p* < 0.001	0.38	C < A^∗∗∗^,B^∗∗∗^
TAS ddf	18.26	4.87	18.78	4.61	11.34	4.00	31.03	*p* < 0.001	0.37	C < A^∗∗∗^,B^∗∗∗^
TAS eot	20.62	5.22	20.40	6.32	16.49	5.35	6.91	*p* = 0.02	0.11	C < A^∗∗^,B^∗^
ER	2.52	0.96	2.09	1.03	3.30	0.77	15.50	*p* < 0.001	0.21	C < A^∗∗∗^,B^∗∗∗^
EC	3.30	1.06	3.34	1.27	4.39	0.79	14.11	*p* < 0.001	0.20	C < A^∗∗∗^,B^∗∗∗^

*^∗^*p* < 0.05, ^∗∗^*p* < 0.01, ^∗∗∗^*p* < 0.001. ^*a*^ = *df* (2,106); ^*b*^ = *df* (2,107). BDI, Beck Depression Inventory; RF, Reflective Function; RME, Reading the Mind in the Eyes; TAS-20, Toronto Alexithymia Scale; TAS dif, difficulty in identifying feelings; TAS ddf, difficulty in describing feelings; TAS eot, externally oriented thinking; ER, Differentiation of Self Inventory – emotional reactivity; EC, Differentiation of Self Inventory – emotional cutoff.*

### Between-Group Differences in Mentalizing and Affect Regulation Patterns

To examine the first hypothesis, we conducted a between-group MANCOVA with age and parents’ education as covariates. The MANCOVA for the five dependent variables— RF, RME, TAS, ER and EC [Wilk’s Lambda *F*_(__10__,__200__)_ = 8.83, *p* < 0.0001]—was significant. The univariate ANCOVAs and the significant between-group contrasts found by the Tukey’s test are presented in Table 2. The data show four significant between-group effects, in RF, *alexithymia* and the two affect regulation patterns, with small to medium effect sizes. Specifically, female adolescents with AN and with depression exhibited lower RF and higher *alexithymia* than controls as well as elevated levels on the specific subscales of the TAS. Both clinical groups reported higher ER and EC than controls. No between-group differences emerged in emotion recognition (RME). Last, no between-group differences in the study variables were found between the two clinical groups.

As the clinical groups did not differ on the BDI score, we further examined the between-group differences by controlling BDI as a covariate, in addition to age and parents’ education. The MANCOVA for the five dependent variables— RF, RME, TAS, ER and EC [Wilk’s Lambda *F*_(__10__,__198__)_ = 2.85, *p* < 0.01]— maintained its significance. Specifically, significant between-group differences were attained for RF [*F*(2,107) = 12.19, *p* < 0.0001, η^2^ = 0.19], TAS [*F*(2,106) = 3.99, *p* < 0.05, η^2^ = 0.07] and the specific emotional subscale of the TAS, ddf [*F*(2,106) = 4.78, *p* < 0.05, η^2^ = 0.08]. In this analysis, the control group differed significantly from the two clinical groups in RF (*p* < 0.001 for depression and *p* < 0.0001 for AN), general TAS (*p* < 0.05) and TAS ddf (*p* < 0.05). Again, no differences were found in these analyses between patients with AN and those with depression. Note that controlling for the BDI score resulted in reducing the effect size of the general TAS and the emotional TAS subscale. Regarding the affect regulation patterns, when controlling for BDI, the between group differences no longer existed [for ER *F*(2,106) = 1.92, NS; for EC *F*(2,106) = 0.07, NS].

### Mentalizing, Affect Regulation Patterns and Depression as Predictors of ED Symptoms

Table 3 presents the Pearson’s Correlations Coefficients between the study measures. In the overall sample, elevated ED symptoms (EAT-26) and elevated depressive symptoms (BDI) were found to correlate significantly with elevated general *alexithymia* and all the specific TAS subscales, as well as with both affect regulation patterns. This data points out that elevated *alexithymia*, and elevated ER and EC, were correlated with both greater depressive symptomatology and greater ED symptomatology. A negative marginally significant correlation was found between RF and ED symptoms. Affect regulation patterns (ER and EC) correlated significantly with elevated general *alexithymia* and all the specific TAS subscales, but not with RF and RME. RF was significantly correlated with overall TAS (*r* = −0.20, *p* < 0.05), and with the specific domain of TAS ddf (*r* = −0.23, *p* < 0.05). RME was not correlated with TAS or with RF.

**TABLE 3:** Pearson partial correlation of mentalization and affect regulation with ED symptoms and depression.

**Variables**	**EAT-26**	**BDI**	**ER**	**EC**
RF	−0.17°	–0.06	0.06	0.04
RME^a^	0.02	–0.03	0.01	0.07
TAS-20^b^	0.52^∗∗∗^	0.71^∗∗∗^	–0.50^∗∗∗^	–0.67^∗∗∗^
TAS dif	0.46^∗∗∗^	0.70^∗∗∗^	–0.54^∗∗∗^	–0.58^∗∗∗^
TAS ddf	0.48^∗∗∗^	0.61^∗∗∗^	–0.39^∗∗∗^	–0.68^∗∗∗^
TAS eot	0.34^∗∗∗^	0.39^∗∗∗^	–0.34^∗∗∗^	–0.34^∗∗∗^
ER	–0.31^∗∗^	–0.51^∗∗∗^	–	–
EC	–0.36^∗∗∗^	–0.57^∗∗∗^	–	–
BDI	0.62^∗∗∗^	–	–	–

*° = 0.07, ^∗∗^*p* < 0.01, ^∗∗∗^*p* < 0.001. ^*a*^*n* = 112; ^*b*^*n* = 113. ED, eating disorder; RF, Reflective Function; RME, Reading the Mind in the Eyes; TAS, The Toronto Alexithymia Scale; TAS dif, difficulty in identifying feelings; TAS ddf, difficulty in describing feelings; TAS eot, externally oriented thinking; ER, Differentiation of Self Inventory – emotional reactivity; EC, Differentiation of Self Inventory – emotional cutoff; EAT-26, Eating Attitude Test 26; BDI, Beck Depression Inventory.*

We conducted three series of hierarchical linear regression analyses to examine hypotheses 2, 3, and 4, in which the severity of the ED symptomatology (EAT-26) served as an outcome measure. The first model included the group of measures assessing mentalizing as predictors (RF and *alexithymia*); the second model included the group of measures assessing emotion regulation patterns, and the third model included both groups of measures and depression in a stepwise regression procedure (see Table 4). Age and parents’ education were included in the first step in each model.

**TABLE 4:** Prediction of ED symptoms by mentalizing variables, affect regulation and depression.

	***Model 1***	***Model 2***	***Model 3***
***Predictors***	**β**	**β**	**β**
Age	0.24^∗∗^	0.13	0.23^∗∗^
Mother’s education	−0.05	0.04	−0.02
Father’s education	−0.18^∗^	−0.27^∗∗^	−0.13
RF	−0.07		−0.10
TAS-20	0.51^∗∗∗^		0.24^o^
EC		−0.26^∗∗^	0.05
ER		−0.14	0.08
BDI			0.49^∗∗∗^
*R*^2^_change_	0.12^**a^,0.25^***b^	0.12^***b^	0.0^c^,0.10^***d^
Δ*R*^2^	0.37^∗∗∗^	0.24^∗∗∗^	0.49^∗∗∗^

**^*o*^* = 0.0503, *^∗^p* < 0.05, *^∗∗^p <* 0.01, *^∗∗∗^p <* 0.001. *^*a*^* = *R^2^ in step 1, ^*b*^R^2^ in step 2, ^*c*^R^2^ in step 3, ^*d*^R^2^ in step 4.* ED, eating disorder; RF, Reflective Function; TAS, Toronto Alexithymia Scale; ER, Differentiation of Self Inventory – emotional reactivity; EC, Differentiation of Self Inventory – emotional cutoff; BDI, Beck Depression Inventory.*

The first model was found to be highly significant, *F*_(__5__,__108__)_ = 13.15, *p* < 0.0001, explaining 37% of the variance in the EAT-26. Specifically, in the first step, age and parents’ education contributed 12% of the variance. The second step comprising of the mentalizing measures block in addition to the control variables was found to be significant *F*_(__5__,__108__)_ = 22.14, *p* < 0.0001, significantly explaining an additional 25% of the variance. The sole significant predictive variable in this block was *alexithymia* (TAS).

The second model, was also found to be significant, *F*_(__5__,__108__)_ = 6.77, *p* < 0.0001, explaining 24% of the variance in the EAT-26. The second step comprising the affect regulation block in addition to the control variables was found to be significant *F*_(__5__,__108__)_ = 8.16, *p* < 0.001, significantly explaining 12% of the variance. The sole significant predictive variable in this block was the *emotional cutoff* pattern (EC).

In the third model, the inclusion of affect regulation patterns in the third step did not have an additional contribution to the explained variance, with TAS as the only significant predicting variable. The addition of the BDI in the fourth step increased the explained variance to 47.8%. In this model, depression was the sole significant variable, and TAS marginally (*p* = 0.0503) predicting greater severity of ED symptomatology.

#### The Mediating Role of Affect Regulation and Depression on Mentalization and ED Symptoms Link

**FIGURE 1:**
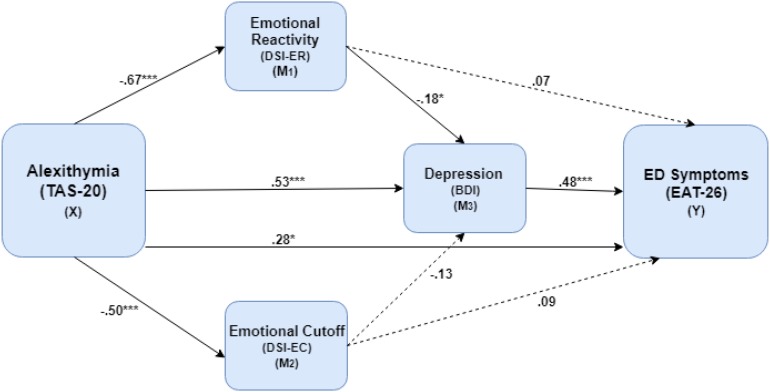
The mediating role of emotional reactivity and depression in the association between Alexithymia level and the severity of ED symptoms. ^∗^*p* < 0.05, ^∗∗∗^*p* < 0.001, ED, eating disorder; TAS-20, Toronto Alexithymia Scale; DSI-ER, Differentiation of Self Inventory – emotional reactivity; DSI-EC, Differentiation of Self Inventory – emotional cutoff; BDI, Beck Depression Inventory; EAT-26, Eating Attitude Test 26.

To examine the fifth hypothesis regarding the mediating role of affect regulation strategies and depressive symptoms between the mentalizing variables (*Alexithymia* and *RF*) and the severity of ED symptoms, we employed two parallel multiple mediation model with TAS or RF scores as independent variables, EAT-26 score as a dependent variable and three potential mediators in the following order: first either ER or EC and second, BDI. Age and parents’ education were included as covariates. Results of the mediation model with TAS as an independent variable are presented in Figure 1.

In this model, the results indicated a serial mediation path with ER and BDI serving as significant mediators (X→M_1_→M_3_→Y) between the TAS and EAT-26 scores (*effect* = 0.04, *CI*_*bootstrap*_: 0.001–0.10). Elevated TAS increased EAT-26 through a negative direct effect on ER [*effect* = −0.67, *SE* = 0.08, *t*(114) = −6.07, *p* < 0.001], negative direct effect of ER on the BDI [*effect* = −0.18, *SE* = 0.08, *t*(114) = −2.34, *p* < 0.001] and, finally, a positive direct effect of BDI on EAT-26 score [*effect* = 0.48, *SE* = 0.11, *t*(114) = 4.38, *p* < 0.001]. Moreover, an indirect effect (X→M_3_→Y) was revealed for the TAS on EAT-26 score through increased BDI score (*effect* = 0.26, *CI*_*bootstrap*_: 0.12–0.45). The direct effect of TAS on the EAT-26 was also significant [*effect* = 0.28, *SE* = 0.12, *t*(114) = 2.38, *p* < 0.05]. These findings indicated that the association between TAS and EAT-26 was partially mediated by ER and BDI. In the second mediation model, with RF as independent variable, no significant mediation paths were found for the association between RF and EAT-26 score.

## Discussion

The current study focused on the dynamics of AN in the framework of the mentalization-based model. Moreover, as there is a substantial comorbidity of depression in patients with AN, we sought to examine whether the deficiencies in mentalizing seen in AN are attributed to the ED *per se*, or alternatively, to comorbid depression. Thus, we compared female adolescent inpatients with AN to female adolescent inpatients with depression and to healthy female adolescent controls.

The first aim of the study was to examine whether the general ability of mentalizing (RF) and specifically, affective mentalizing regarding self and others are related to AN and depressive symptoms. For this purpose, we used the constructs of *alexithymia* and *ToM* to explore the specific dimension of affective mentalizing regarding self vs. others, rather than examining only the general ability of RF. The underlying assumption was that the two clinical groups would exhibit lower RF, lower ability to identify the emotions of others (*ToM*), and greater *alexithymia* than the control group. We further anticipated that patients with AN would exhibit a greater deficiency in mentalizing affective experience of self (*alexithymia*) than patients with depression, whereas participants with depression would exhibit a lower capacity for mentalizing of the other (*ToM*) than patients with AN.

Another aim was to examine affect regulation patterns in the three groups, their predictive ability of ED symptoms beyond that of the mentalizing variables, and their potential role in mediating between mentalizing and ED symptoms. We hypothesized that the two clinical groups would similarly report deficient affect regulation patterns compared to controls and that affect regulation patterns will have additive contribution to the prediction of ED symptoms beyond the mentalizing variables. We also hypothesized that affect regulation patterns will serve as mediating factors between mentalization and depressive and ED symptoms.

### Between-Groups Differences in Mentalizing and Affect Regulation Patterns

The first hypothesis anticipating between-group differences in mentalizing and affect regulation measures was mostly confirmed. Thus, participants with EDs and depression exhibited lower levels of general RF, higher levels of *alexithymia* and deficient affect regulation patterns than controls. Contrary to our hypothesis, the two clinical groups did not differ on the self-other pole of mentalizing.

In addition, the differences between the two clinical groups and controls in RF, general TAS and the ddf subscale of the TAS, were still present after controlling for the reported depressive symptoms (BDI). Nonetheless, after controlling for BDI, the effect size of the between-group differences in *alexithymia* was quite small. The findings regarding alexithymia are in line with previous studies showing an association between *alexithymia* and depression (Taylor and Bagby, 2004).

The data regarding RF, which still differentiates between the clinical and control groups even after controlling for BDI, and the lack of correlation between RF and BDI, may point that contrary to alexithymia, a deficiency in general mentalizing ability is a transdiagnostic deficiency, not confounded by depressive symptoms.

The lack of differences in affect regulation patterns between patients with AN and depression, may suggest that impaired emotion regulation may represent a common predisposing or maintaining mechanism for both disorders (Donofry et al., 2016). Indeed, depression and EDs have been found to share structural and functional alterations in brain regions involved in emotion regulation, including the amygdala, ventral striatum, nucleus accumbens, anterior cingulate cortex, insula, and dorso-lateral prefrontal cortex (Donofry et al., 2016). There is a suggestion that deficiencies in affect regulation may represent a transdiagnostic risk or maintenance factor not only in EDs or depression, but rather in psychopathology in general (e.g., Svaldi et al., 2012). Nonetheless, our finding that after controlling for BDI, the clinical groups do not differ from the control group in affect regulation, suggest that it is the depression that actually increases deficient affect regulation in AN in terms of affective constriction and inhibited emotional expression. These findings stand in contrast to studies suggesting that inhibited over-controlled affective response may be considered a core trait of AN above and beyond the influence of depression (Anderluh et al., 2009).

The lack of between-group differences in RME in our study contrasts with previous studies showing differences in RME between patients with AN (Harrison et al., 2010) and depression compared to controls (Demenescu et al., 2010). Nonetheless, other studies have found no deficits in emotion identification in patients with AN in comparison to controls (Bentz et al., 2017). Future study of deficiencies in emotion recognition may be better detected when examined in a more naturalistic social context, such as the Movie for the Assessment of Social Cognition instrument (Dziobek et al., 2006), than when assessed under static conditions, such as those introduced in our design.

Furthermore, our data show that patients with AN differ from the control group in mentalizing regarding the self (*alexithymia*) but not in mentalizing the experiences of others (RME). This self/other gap in patients with AN is in accordance with the findings of Skårderud and Fonagy (2012), suggesting that people with EDs do not develop a coherent sense of self from within. Rather, they invest in acquiring a sense of self based on the reactions of others. This discrepancy further corresponds with self-psychology conceptualizations, namely that the development and maintenance of an ED may be associated with attunement to the needs of the other, at the cost of relinquishing one’s own wishes (Bachar et al., 2010).

### Mentalizing, Affect Regulation Patterns and Depression as Predictors of ED Symptoms

The second, third, and fourth hypotheses were partially confirmed. The preliminary data showed significant correlations between ED symptoms and *alexithymia and* affect regulation patterns, and a marginally significant correlation with RF. Yet, a more detailed examination showed that for the mentalizing block, *alexithymia* was the sole variable predicting the severity of ED symptoms, i.e., elevated *alexithymia* predicted more severe ED symptoms. The lack of a significant contribution from RF may be related in part to the common variance between *alexithymia* and RF found in our study, and to the stronger ability of affective mentalizing regarding the self (TAS) to predict ED symptoms than general reflective functioning.

Upon the introduction of depression into the regression model, *alexithymia* still, but only marginally significantly, contributed to the severity of ED symptoms. Although the common variance between *alexithymia* and depression found in our study and elsewhere in patients with AN (Corcos et al., 2000), that led to attenuation of the predictive value of alexithymia, we still found that the TAS has a distinctive contribution to the severity of ED symptoms.

Both affect regulation patterns were significantly correlated with ED symptoms, but the sole significant predictor of ED symptoms was the EC pattern. Previous studies found that suppression and deactivation of affects were related with ED symptoms (Rothschild-Yakar et al., 2018). Nonetheless, in the present study we found an indirect influence of ER on the severity of ED symptoms via attenuation of depressive symptoms. Altogether, these finding suggests that both seemingly contrasting affect regulation patterns may contribute to the severity of AN as was suggested by a systematic review and meta-analysis of self-report data (Oldershaw et al., 2015) Our study adds to the field by pointing that affect regulation deficiencies are mainly related to depressive component in AN.

The correlation found between both affect regulation patterns and the TAS, is in accordance with the inclination of individuals with alexithymia to use more suppressive and less reappraisal strategies (Swart et al., 2009) and with the notion that identifying one’s feelings accurately and communicating them to others plays a role in the self-regulation of distressing emotional states (Taylor et al., 1999). Whereas alexithymia mainly focuses on affects regarding the self, the general mentalizing capacity of RF relates to affective and cognitive aspects regarding both self and others. Thus, the lack of significant correlation between RF and affect regulation patterns may point out that affect regulation in the study groups is mainly related with affective mentalizing, and that affect regulation patterns and general mentalizing are distinct processes.

### The Mediating Role of Affect Regulation and Depression on the Link Between Mentalization and Severity of ED Symptoms

Affective mentalizing as examined with the alexithymia construct was found in our study as directly accounting for elevated ED symptoms, and also as indirectly connected with ED symptoms via elevated emotional hyperactivation (ER) and elevated depressive symptoms. Thus, reviewing the role of alexithymia in EDs raises the question whether it is a specific predisposing and maintaining factor in EDs, or whether it is confounded with depression (due to the inconclusive results found when controlling for the depressive symptoms) (Westwood et al., 2017). Our study comparing patients with AN and with depression suggests that alexithymia is a transdiagnostic trait, elevated in both disorders. Nonetheless, the results of the mediating model, the predictive ability of TAS alongside the depressive symptoms, and the significant difference in TAS between AN patients and controls found even after controlling for depressive symptoms, may all point out that alexithymia is a core personality trait in AN, above and beyond the influence of depression.

The data that the AN group differed from the controls in general mentalizing ability (RF), and that the RF was marginally significantly correlated with ED but not with depressive symptoms suggests a connection between general mentalizing and AN. Nonetheless, there was no significant direct or indirect role of general mentalization in predicting the severity of ED symptoms. Further research should explore the role of general mentalizing in the predisposition and maintenance of AN and in the depression-AN link.

## Limitations, Conclusion, and Recommendations for Future Research

The findings of this study should be interpreted in light of its limitations. First, the group of patients with depression was relatively small, so that we were not able to assess the influence of the patients’ comorbid psychiatric disorders on the findings. Second, the presence, or absence, of psychiatric disorders was determined differently in the three groups. Moreover, whereas weight and height were assessed in the two research groups, these measures were obtained by self-report among the controls. Third, both clinical groups received anti-depressive medications when assessed, which could have influenced the findings. Nonetheless, despite being treated, both patient groups were more depressed than the controls. Fourth, our research design was cross-sectional, allowing only for inferences about associations but not causality. Fifth, the age range of the three groups was wide. Since maturation effects might influence the study measures such as mentalizing abilities, future study should examine these potential trends in adolescents compared to young adults. Last, as our clinical samples included only inpatients, our findings cannot be generalized to outpatients with less severe AN and depression.

Despite these limitations, our study has several important contributions. To the best of our knowledge, it is the first to compare RF in patients with AN to patients with depression and not only to healthy controls. The lack of differences between the clinical groups in deficiencies in general mentalizing and *alexithymia* suggests that these deficiencies may not be unique to AN (or to depression). Rather, they may represent a transdiagnostic deficiency. Our data may support models suggesting the existence of a general psychopathology factor (a P factor) in psychiatric disorders (Caspi et al., 2014). This approach points out the importance of promoting mentalizing ability and affect awareness in the treatment of various psychopathologies.

The role of mentalization variables, specifically alexithymia in predicting the severity of ED symptoms, alongside the predictive value of *emotional cutoff* and the mediating role of *emotional reactivity* in attenuating depressive symptoms, may have clinical implications. Thus, treatment of EDs should focus not only on the behavioral aspects of the disorder and the comorbid depressive symptoms, but also should assist the patients in improving their ability to think reflectively and specifically to identify and express their emotions (Skårderud and Fonagy, 2012), alongside improving their ability to adequately cope with their emotions.

### Summary and Direction for Future Research

Our findings show that patients with AN and depression exhibited lower levels of RF, higher levels of *alexithymia*, and reported deficient affect regulation patterns vs. controls, whereas no between-group difference was found in *ToM*. The two clinical groups did not differ in any of these variables. Second, elevated *alexithymia* and depressive symptomatology, but not mentalizing, predicted more severe ED symptomatology. Third, alexithymia directly accounted for elevated ED symptoms and also indirectly connected with ED symptoms via elevated ER and elevated depressive symptoms.

Future large-scale studies of ambulatory samples with different types of EDs and different psychopathological disorders, using other tools for the assessment of mentalization and affect recognition and regulation may widen the scope of our preliminary findings and explore other factors potentially differentiating between patients with EDs and other psychopathologies. For example, a specific developmental level in mentalizing self-emotional experience can be compared to metalizing the experience of the other by using the performance-based measure of the Levels of Emotional Awareness Scale (LEAS, Lane et al., 1990). This scale evaluates the individual’s capacity to describe self-emotional experiences and also the emotional states of others.

Last, longitudinal prospective studies examining mentalizing from the onset of the illness to recovery may point out to whether deficient mentalizing at the acute state of the illness represents a collapse and a state correlate, or rather a core developmental trait potentially associated with the outcome of AN, depression, and other psychopathologies.

## Data Availability Statement

The datasets generated for this study are available on request to the corresponding author.

## Ethics Statement

The studies involving human participants were reviewed and approved by the Internal Review Boards (Helsinki Boards) of the Sheba Medical Center, Tel Hashomer, and the Geha Mental Health Center, Petah Tikva, Israel. Written informed consent to participate in this study was provided by the participants’, and their legal guardian/next of kin where appropriate.

## Author Contributions

LR-Y and DS substantially contributed to all phases including conception and design, analysis and interpretation of data, and writing and final approval. DG, GS, AY, GE, BK, and EG contributed to the administration, analysis, interpretation of data, and critical revision. All the authors approved the article for publication.

## Conflict of Interest

The authors declare that the research was conducted in the absence of any commercial or financial relationships that could be construed as a potential conflict of interest.
